# Mortality probabilities after revascularization and medical therapy in CAD patients under 60 years old: a meta-analysis study

**DOI:** 10.1186/s43044-021-00225-x

**Published:** 2021-11-04

**Authors:** Mohammad Afrouzi, Farbod Ebadi Fard Azar, Ali Aboutorabi, Marjan Hajahmadi, Seyed Javad Ebadi

**Affiliations:** 1 Department of Health Economics, School of Health Management and Information Sciences, Iran University of Medical Sciences, Tehran, Iran; 2 Health Promotion Research Center, Iran University of Medical Sciences, Tehran, Iran; 3 Cardiovascular Department, Rasoul Akram General Hospital, Iran University of Medical Sciences, Tehran, Iran; 4 Qazvin Army Hospital 553, Qazvin, Iran

**Keywords:** Coronary artery disease, Death probabilities, Revascularization, Medical therapy

## Abstract

To estimate death probabilities after coronary artery bypass graft (CABG), percutaneous coronary intervention (PCI), and medical therapy (MT) in patients under 60 years old. We conducted a search systematic on PubMed, Embase, Cochrane Library, and Web of Science up to January 2021. The study included three parts. In the probabilities part (A), Comprehensive Meta-Analysis, and in the comparison parts (B and C), Review Manager was used in conducting meta-analyses. Nine studies consisting of 16,410 people with a mean age of 51.2 ± 6 years were included in the meta-analysis. Over a mean follow-up of 3.7 ± 2 years, overall mortality after CABG, PCI and MT was 3.6% (95% CI 0.021–0.061), 4.3% (95% CI 0.023–0.080) and 9.7% (95% CI 0.036–0.235), respectively. The length of follow-up periods was almost the same and did not differ much (*p* = 0.19). In Part B (without adjustment of baseline characteristics), 495 (4.0%) of 12,198 patients assigned to CABG died compared with 748 (4.5%) of 16,458 patients assigned to PCI (risk ratio [RR]: 0.77, 95% CI 0.50–1.20; *p* = 0.25). Seventy-four (3.5%) of 2120 patients assigned to CABG and 68 (4.2%) of 1621 patients assigned to PCI died compared with 103 (9.5%) of 1093 patients assigned to MT in equal follow-up periods (CABG-MT: RR 0.34; 95% CI 0.23–0.51; *p* < 0.002) (PCI-MT: RR 0.40; 95% CI 0.30–0.53; *p* = 0.02). In Part C, overall mortality after PCI in PACD patients with STEMI was higher in elderly versus young (RR 2.64; 95% CI 2.11–3.30) and is lower in men versus women (RR 0.61; 95% CI 0.44–0.83). Mortality probabilities obtained are one of the most important factors of effectiveness in the economic evaluation studies; these rates can be used to determine the cost-effectiveness of procedures in CAD patients aged < 60 years.

## Background

Coronary artery disease is the leading cause of death among patients, and its management continues to be a major clinical challenge [1]. Patients with symptoms from flow-limiting atherosclerotic coronary artery narrowing can be managed with coronary artery bypass graft (CABG), percutaneous coronary intervention (PCI), medical therapy (MT), or combinations of all strategies [1, 2]. The age range of 20–60 years is very close to the PCAD age range, as in most literature works, premature coronary artery disease (PCAD) is defined as the occurrence of an acute myocardial infarction (MI) or a symptomatic myocardial ischemia with an obstructive coronary artery disease (stenosis 70%) before age 55. It is usually linked with history of premature CAD in the family [1–8].

The first and key step in starting an evaluation of treatments is to determine the effectiveness of treatments [3]. The effectiveness of treatments in young patient with coronary artery disease is very important from social and economic aspects [4]. The most important element in measuring of effectiveness is probability of survival; which is directly related to probabilities of death [5].

One of the major meta-analyses in 2009 comparing CABG with PCI reported occurrences of death, myocardial infarction, and repeat revascularization from ten randomized trials [6]. In this study, 107 (10%) of 1063 patients younger than 55 years who were assigned to CABG died compared with 88 (8%) of 1122 patients assigned to PCI. There is not much mention of outcomes of PCAD patients’ especially. Also, previous meta-analyses did not determine the common treatment strategies in PCAD and the effectiveness of treatment for CAD patients under 60 years old remains controversial [7–12].

The present study aimed to address this deficiency and determine the mortality rate after CABG, PCI and MT in CAD patients under 60 years old. The obtained probabilities will be used in economic evaluation study such as cost-effectiveness of procedures.

The analysis was performed in three parts: In the first part (Part A) that is the main part, the probabilities were determined separately for each procedure. Our goal in this part was to determine the probability values of each of the decision branches (CABG, PCI, and MT) in the decision tree of the Markov model. We are going to do an economic evaluation based on these two scenarios: Determining the cost-effectiveness of treatments based on the probabilities of endemic mortality (Scenario 1) and the probabilities of international mortality (Scenario 2). In the second and third parts (Part B and C) that is the side part of the study, comparisons were made based on the type of treatment or the type of baseline characteristic such as STEMI.

The study protocol was registered in the International Prospective Register of Systematic Reviews (PROSPERO) with the registration number of CRD42020189837. We used the Preferred Reporting Items for Systematic Reviews and Meta-Analyses (PRISMA) checklist when writing this report [13].

PubMed, Embase, Cochrane Library, and Web of Science were searched up to January 2021 in order to find relevant literature works. In addition, the reference lists of final clinical trials and review articles were scanned to find additional records. The search terms were “Coronary artery disease,” “Coronary artery bypass grafting,” “Percutaneous coronary intervention,” “Medical therapy,” “outcomes” and “adverse events.”

After identifying and elimination of duplicate studies, the two reviewers (HM&EF) independently were screened the titles and abstracts and full text of included records. Disagreement between reviewers was solved by discussion. Studies with the conditions of the following cases considered for inclusion:

- The minimum and maximum age of patients with CAD should be 20 and 60 years, respectively. (In case of insufficient studies related to a procedure such as MT, the maximum age will be increased to 65 years, exceptionally)
- Patients who are undergoing at least one of the CABG, PCI, and MT treatments
- Patients were followed for at least 1 year
- Patients did not undergo other treatments.
  The exclusion criteria were as follows:
- studies that examined short-term outcomes such as in-hospital mortality
- studies without crud rate of mortality
- studies that have not included death in the end points

The CONSORT and STROBE checklists were used to assess the methodological quality of randomized control trial and cohort studies, respectively. Two authors (AM&AA) independently were extracted the data using the same extraction from including (a) patients’ characteristics (sex, age, number, and place), (b) treatment interventions (CABG, PCI, MT), (c) outcomes (mortality, MI …). The two reviewer checked the titles and abstracts independently. After identifying and elimination of duplicate studies, two reviewer divided them into two related and unrelated groups. Then, the team of authors reviewed the full text of two reviewer for the final selection. Finally, nine studies were selected [14–22]. Table 4 lists the characteristics of the studies, included year, country, design, comparison groups, etc.

The meta-analysis was conducted using Comprehensive Meta-Analysis version V2 and Review Manager (RevMan) version 5.1 software. Risk ratio with a 95% confidence interval (CI) was used for dichotomous variables. Statistical heterogeneity was evaluated using I-square > 50%, and Chi-square with a significance level *p* < 0.1 was used to assess the statistical heterogeneity. The random-effects method was used for statistical heterogeneity; otherwise, the fixed-effect method was used.

## Main text

**Fig. 1:**
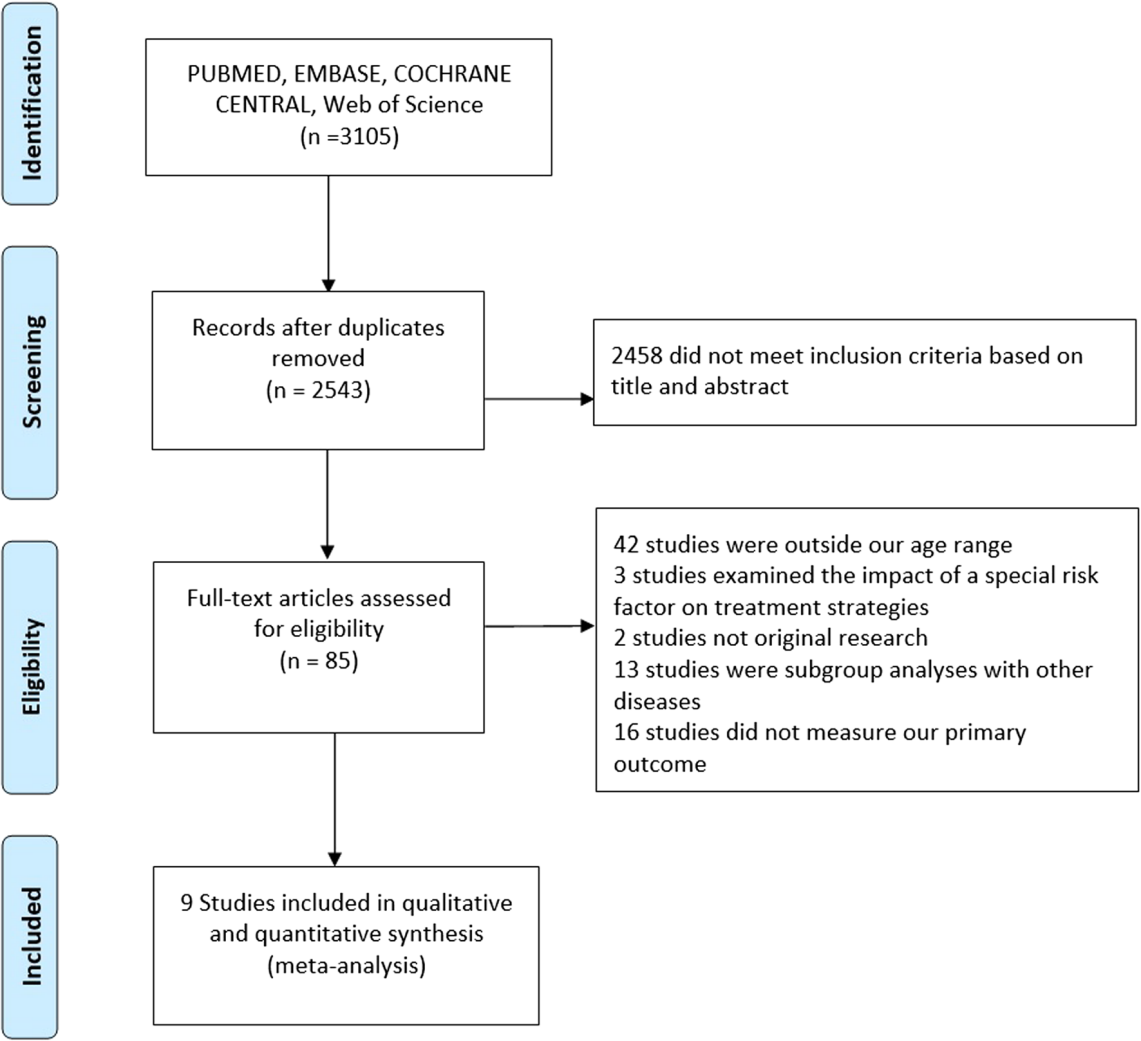
Flowchart of selected studies

Fig. 1 shows the process of literature searching, removing, and screening and the records. Nine studies, including 7 cohorts and 2 randomized clinical trial, with 16,410 patients were selected. 2,665 (16.3%) patients were female and the mean age of the study population was 51.2 ± 6 years that varied from 39.6 to 53.8 years in eligible studies. Baseline characteristics of patients are presented in Table 1, and characteristics of studies are presented in Table 4.

**Table 1:** Baseline characteristics of CAD patients under 60 years old

Study	Overall (N = 16,410)	STICH (n = 625)	LOTUS (n = 1792)	Li et al. (n = 2018)	COURAGE (n = 1381)	Rosato et al. (n = 2547)	Otten et al. (n = 3714)	Rathod et al. (n = 367)	Roth et al. (n = 2046)	Fu et al. (n = 1920)
Age (years)	51.2 ± 6	53.3 ± 6	47.7 ± 6	41.6 ± 3	55.5 ± 6	53.8 ± 4	54.2 ± 6	39.6 ± 5	52.0 ± 5	52.6 ± 7
Female	2665 (16.2%)	61 (9.8%)	486 (27.1%)	142 (7.0%)	184 (13.3%)	265 (10.4%)	708 (19.1%)	47 (12.8%)	545 (26.6%)	227 (11.8%)
Family history	4241/13238 (32.0%)	NA	700 (39.1%)	268 (13.3%)	766 (55.5%)	NA	1775 (47.8%)	122 (33.2%)	418 (20.4%)	192 (10.0%)
History of MI	3160/15785 (20.0%)	479 (76%)	606 (33.8%)	498 (24.7%)	550 (39.8%)	718 (28.2%)	262 (7.1%)	26 (7.1%)	329 (16.1%)	171 (8.9%)
History of PCI	1383/13863 (10.0%)	83 (13.3%)	236 (13.2%)	291 (14.4%)	214 (15.5%)	NA	181 (4.9%)	22 (6.0%)	225 (11.0%)	131 (6.8%)
History of CABG	329/12472 (2.6%)	18 (2.9%)	37 (2.1%)	NA	111 (8.0%)	36 (1.4%)	67 (1.8%)	0 (0.0%)	60 (2.9%)	NA
History of stroke	493/12472 (3.9%)	37 (5.9%)	92 (5.1%)	61 (3.0%)	NA	48 (1.9%)	67 (1.8%)	NA	NA	188 (9.8%)
Diabetes mellitus	2965/14392 (20.6%)	224 (35.8%)	618 (34.5%)	2018 (100%)	456 (33.0%)	675 (26.5%)	305 (8.2%)	36 (9.8%)	301 (14.7%)	350 (18.2%)
Hypertension	6604/13863 (47.6%)	355 (56.8%)	1116 (62.3%)	1198 (59.4%)	884 (64.0%)	NA	1044 (28.1%)	81 (22.1%)	1027 (50.2%)	899 (46.8%)
Hyperlipidemia	4122/10464 (39.4%)	364 (58.2%)	1091 (60.9%)	NA	NA	NA	838 (22.6%)	80 (21.8%)	1171 (57.2%)	578 (30.1%)
Current smoker	7548/13863 (54.4%)	168 (26.9%)	1033 (57.6%)	1413 (70.0%)	548 (39.7%)	NA	2234 (60.2%)	230 (62.7%)	556 (27.2%)	1366 (71.1%)
BMI (kg/m2)	27.7 ± 2.5	27 ± 3.5	26.5 ± 3.2	27.4 ± 3.3	30 ± 5.0	NA	NA	NA	27.7 ± 5.0	NA
LVEF (%)	**–**	28 (22, 34)	59.4 ± 13.1	59.3 ± 9.5	61.0 ± 11	NA	NA	45.3 ± 7.3	60 (47_69)	NA
Special clinical characteristic	**–**	HF-LVSD	TVD	DM	Stable angina	NA	STEMI	STEMI	Stable angina	STEMI
TVD	4891/11220 (43.6%)	360 (57.6%)	1792 (100%)	NA	381 (27.6%)	NA	1578 (42.5%)	81 (22.1%)	393 (19.2%)	666 (34.7%)

PCI: percutaneous coronary intervention; CABG: coronary artery bypass graft; MT: medical treatment; NA: not available; LVEF: left ventricular ejection fraction; TVD: three vessel disease; BMI: body mass index; HF-LVSD: heart failure and left ventricular systolic dysfunction; DM: diabetes mellitus; STEMI: ST-elevation myocardial infarction

### Death probabilities

Table 2 presents the main findings. Overall mortality, regardless of the type of treatment, was 4.7% (95% CI 0.033–0.067) during 3.7 ± 2 years of follow-up. Over a mean follow-ups of 4.3 ± 2.9, 3.5 ± 2.8 and 5.4 ± 1.6 years, the overall mortality rates of CABG, PCI and MT were 3.6% (95% CI 0.021–0.061), 4.3% (95% CI 0.023–0.080) and 9.7% (95% CI 0.036–0.235), respectively.

**Table 2:** Death probabilities following CABG, PCI and MT in CAD patients under 60 years old

Treatments	Number of patients	Age (year)	Follow-up (year)	Mortality rate (%)	Lower and upper limit	Z-value
CABG	3523	52.2 ± 6	4.3 ± 3	3.6	0.021–0.061	− 11.39
PCI	11,169	49.0 ± 7	3.5 ± 3	4.3	0.023–0.080	− 9.39
MT	1093	53.1 ± 6	5.4 ± 2	9.7	0.036–0.235	− 4.162
Overall	15,785	51.1 ± 6	3.7 ± 3	4.7	0.033–0.067	− 15.716

CABG: Coronary Artery Bypass Grafting; PCI: Percutaneous Coronary Intervention; MT: Medical Therapy

Also, over a mean follow-up of 3.4 ± 2.5 years, the overall mortality rate of revascularization (n = 14,692) was 3.9% (95% CI 0.027–0.057). These probabilities were estimated 2.2% (95% CI 0.015–0.032), 3.6% (95% CI 0.031–0.042(, 4.4% (95% CI 0.025–0.077), and 5.3% (95% CI 0.037–0.077) during 1, 3, 5 and 7 years of follow-up, respectively (Table 3).

**Table 3:** Death probabilities after revascularization (CABG or PCI) in CAD patients under 60 years old at different follow-ups

Studies	Follow-up (year)	Mortality rate (%)	Lower and upper limit	Z-value
Li, Rosato, Otten, Rathod, Fu	1	2.2	0.015–0.032	− 19.329
Rosato, Rathod, Fu	3	3.6	0.031–0.042	− 42.354
Li, Rosato, Roth, COURAGE	5	4.4	0.024–0.077	− 10.105
LOTUS, Rosato	7	5.3	0.037–0.077	− 14.318
Overall	3.4 ± 2.5	3.9	0.027–0.057	− 16.119

**Fig. 2:**
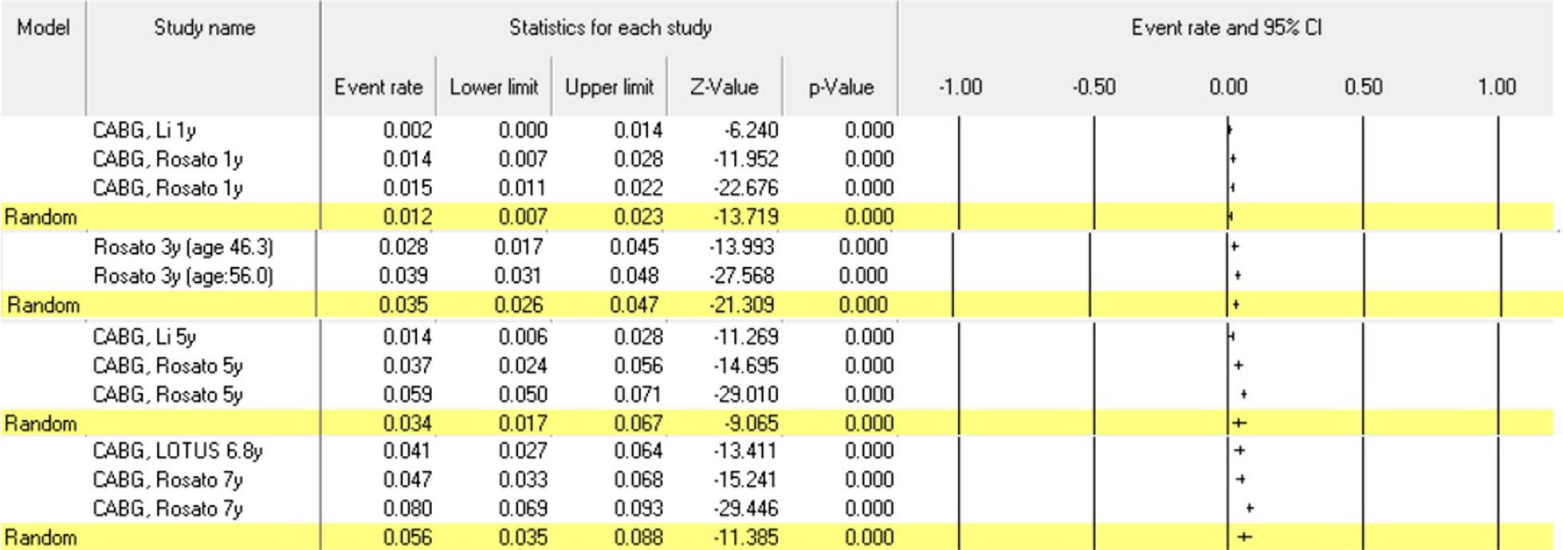
Death probabilities after CABG in CAD patients under 60 years old at different follow-ups

**Fig. 3:**
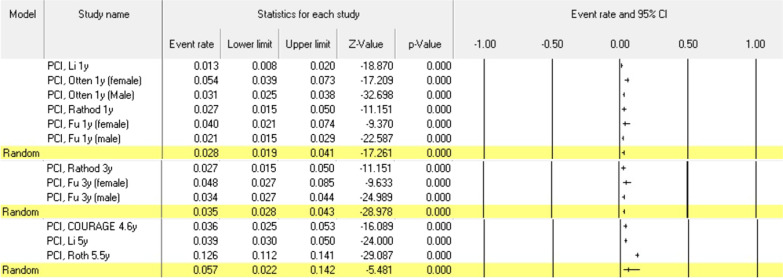
Death probabilities after PCI in CAD patients under 60 years old at different follow-ups

These values after CABG (n = 3523) were 1.2% (95% CI 0.007–0.023), 3.5% (95% CI 0.026–0.047), 3.4% (95% CI 0.017–0.067), and 5.6% (95% CI 0.035–0.088) in 1, 3, 5 and 7 years of follow-up, respectively (Fig. 2). These values after PCI (n = 11,169) were 2.8% (95% CI 0.019–0.041), 3.5% (95% CI 0.028–0.043), 5.7% (95% CI 0.022–0.142) in 1, 3 and 5 years of follow-up, respectively (Fig. 3).

**Fig. 4:**
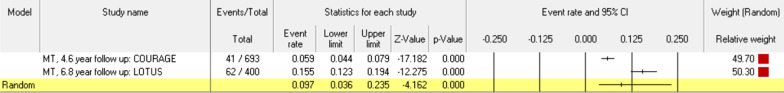
Death probabilities after MT in CAD patients under 60 years old during 5.4 ± 2 years of follow-up

For MT (n = 1093), it was not possible to differentiate the probability of death based on the different follow-ups; because there were only two studies and each study had one follow-up period. However, during 5.4 ± 2 years, mortality rate of MT was 9.7% (95% CI 0.036–0.0235) (Fig. 4).

In this part, Egger test was used to investigate the presence of diffusion bias. The results showed that despite the asymmetric funnel diagram, there was no diffusion bias in any of the treatments (*p* value: CABG = 0.11, PCI = 0.49 and MT = 0.51). In order to ensure the absence of diffusion bias, the method of arrangement and completion was used. In this method, studies that appear to have been omitted were placed in a funnel diagram, and estimates of overall effects were reported in the non-placement and placement mode. The results showed that there is no change in the estimation of the stochastic effects model; so that, in both cases, the probabilities obtained are equal, that confirm the Egger test. To ensure more, random effects model was used to estimate the overall mortality probabilities for all treatments. Because according to the I2 index, the studies have significant heterogeneity; 95.4% (Tau^2^ = 0.295, *p* value < 0.001), 59.7% (Tau^2^ = 0.062, *p* value = 0.041) and 96.0% (Tau^2^ = 0.74, *p* value  < 0.001) for CABG, PCI and MT, respectively.

### Comparison of procedures (without adjustment)

**Fig. 5:**
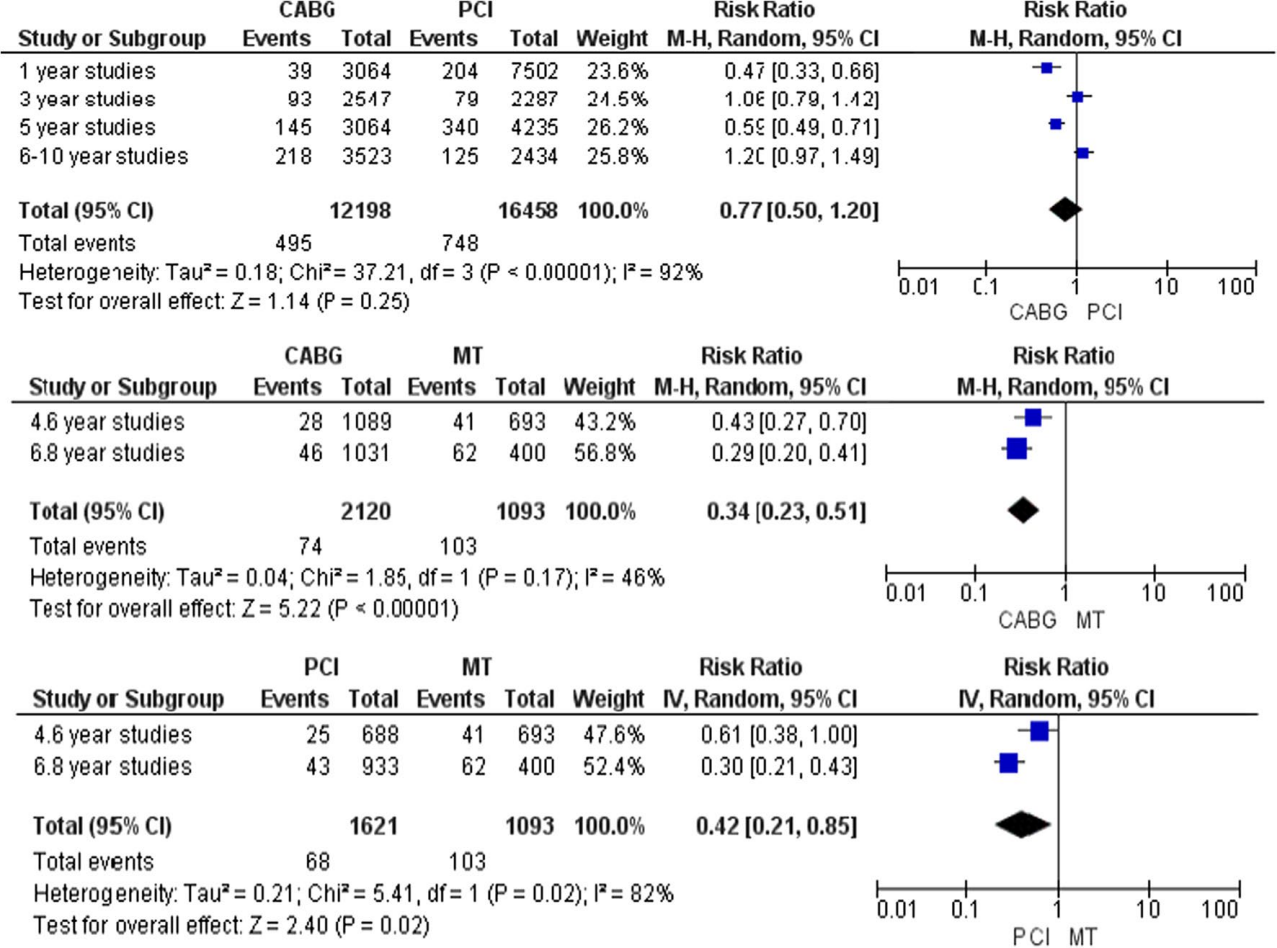
Comparisons of procedures in CAD patients under 60 years old without adjustment of baseline characteristics

Figure 5 presents the comparisons of procedures without adjustment for baseline characteristics. Over a mean follow-up of 3.8 ± 3 years, 495 (4.1%) of 12,198 patients assigned to CABG died compared with 748 (4.5%) of 16,458 patients assigned to PCI. Regardless of the diagnostic-clinical characteristics, no significant difference was observed between CABG and PCI (RR: 0.77, 95% CI: 0.50, 1.20, *p* = 0.25). Also, over a mean follow-up of 5.9 ± 2 years, 74 (3.5%) of 2120 patients assigned to CABG died compared with 103 (9.5%) of 1093 patients assigned to MT. Significant difference was observed between CABG and MT (risk ratio [RR] 0·34, 95% CI 0·23–0·51; *p* < 0.0001). Also, over a mean follow-up of 5.6 ± 1 years, 68 (4.2%) of 1621 patients assigned to PCI died compared with 103 (9.5%) of 1093 patients assigned to MT. Significant difference was observed between PCI and MT (risk ratio [RR] 0·42, 95% CI 0·21–0·85; *p* < 0.0001).

We made comparisons by random effects. Even based on this model, it cannot be said with certainty that events are different between the two treatments because the heterogeneity coefficient was very high (I2 > 75%). The reason may be due to the difference in clinical characteristics of patients in studies with 1 and 5 years follow-up from studies with 3 and more than 6 years follow-up.

### Comparisons based on baseline characteristics

**Fig. 6:**
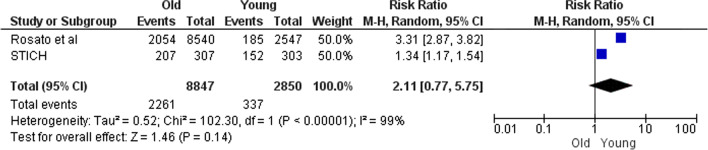
Death after CABG in old versus young

**Fig. 7:**
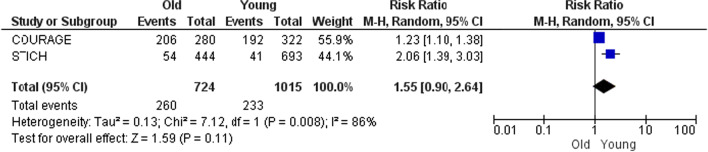
Death after MT in old versus young

In this part, comparisons were made based on special characteristics such as age, sex, and STEMI. Figures 6 and 7 show that following CABG overall mortality does not differ significantly between old and young population (RR 2.11; 95% CI 1.17–1.54). The same is true after MT (RR 1.55; 95% CI 0.90–2.64).

**Fig. 8:**
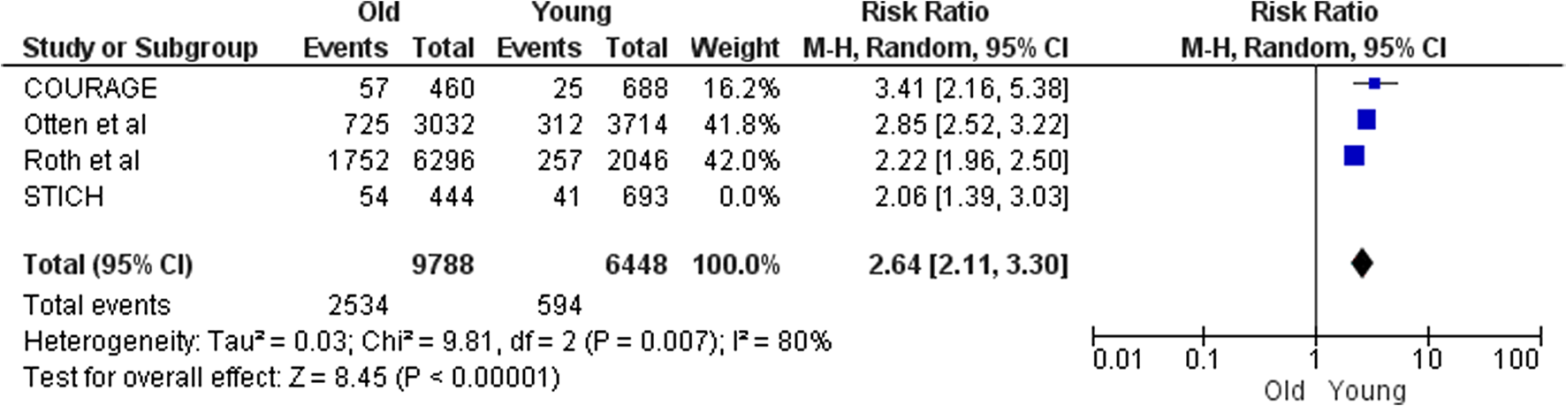
Death after PCI in old versus young

**Fig. 9:**
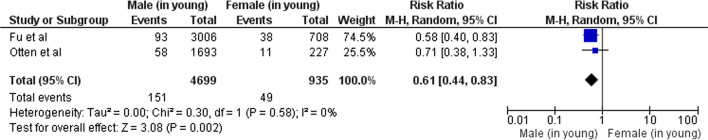
Death after PCI in male versus female under 60 years old and with STEMI

Figures 8 and 9 show that following PCI overall mortality was significantly different based on age and sex. It was observed that overall mortality after PCI in PACD patients with STEMI was much higher in elderly versus young (RR 2.64; 95% CI 2.11–3.30) and is lower in men versus women (RR 0.61; 95% CI 0.44–0.83).

**Fig. 10:**
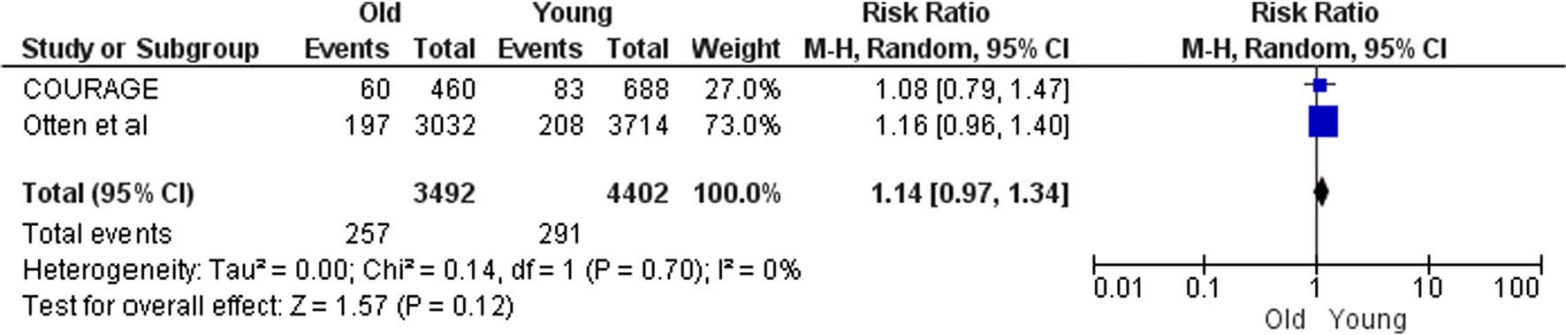
Myocardial infraction after PCI in old versus young

**Fig. 11:**
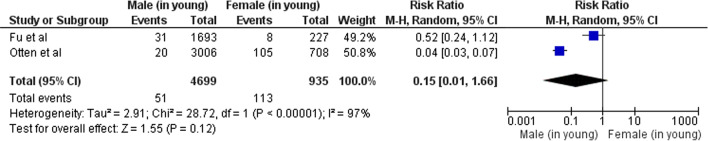
Myocardial infraction after PCI in male versus female under 60 years old and with STEMI

Regarding the outcome of myocardial infarction following PCI, a significant difference was observed between the elderly and young people (RR 1.14; 95% CI 0.97–1.34); and also males compared to females (RR 0.15; 95% CI 0.01–1.66). (Figs. 10, 11, respectively).

## Conclusions

Clinical trial and cohort studies provide suitable information on the effectiveness of treatments. The effectiveness among patients in studies may have differences and similarities [14–22]. The results of a single study are not sufficient to make a decision. There are statistical limitations and clinical differences [2]. This meta-analysis was performed to provide the probability of the overall mortality following CABG, PCI and, MT in CAD patients under 60 years old. The age range of this study (20–60 years old) is very close to the PCAD age range. As in most literature works, premature coronary artery disease (PCAD) is defined as the occurrence of an acute myocardial infarction (MI) or a symptomatic myocardial ischemia with an obstructive coronary artery disease (stenosis 70%) before age 55 [6, 14, 21].

In part A, the main goal which is generally the main purpose of the study was to determine the mortality probabilities after procedures to insert in the decision tree of the Markov model in a cost-effectiveness study. Our main purpose is not to compare the effectiveness of procedures based on mortality; so there is no need to homogenize and adjusting of populations based on clinical-diagnostic characteristics. Because the main goal is to determine the probability of death after procedures to determine their cost-effectiveness in a separate study. Also, in the cost-effectiveness study, which will subsequently use these findings, the main purpose is only to determine the cost-effectiveness of the procedures (CABG, PCI and, MT), not comparing cost-effectiveness (Instead of comparing cost-effectiveness).

The main results of meta-analysis (in part A) showed that the CABG, PCI and MT have 3.6%, 4.3% and 9.7% mortality during 4.3, 3.5 and 5.4 years of follow-up, respectively. Diabetic and multi-vessel patients play an important role for the death probability after CABG. Because it was obtained from three studies. The full population of one of the studies (Li et al.) consisted of diabetic and another study (LOTOS) consisted of TVD patients [14, 21]. But for death probability after PCI, two other factors played a more important role. Its death probability was obtained from six studies. The full population of the three studies consisting of STEMI patients and the full population of two other studies consisting of stable angina patients. Therefore, the probability of death after PCI is more related to STEMI and stable angina. The MT population also did not have a specific feature. This enhances the generalizability of death probability following MT (Tables 4, 5).

**Table 4:** Characteristics of included studies

Author (study name), year	Country	Design	Patients (n, female)	Intervention (characteristic)	Control	Follow-up
Teo et al. (COURAGE), 2009	USA	Randomized	1381, 184	PCI versus MT (Stable angina)	In young versus old	4.6 year
Petrie et al. (STICH), 2016	USA	Randomized	625, 61	CABG versus MT (HF-LVSD)	In different ages	9.8 year
Rosato et al., 2015	Italy	Matched multicenter cohort (prospective)	2547, 265	CABG	In different ages	6.0 year
Rathod et al., 2015	UK	Single center cohort (retrospective)	367, 47	PCI (Primary)	In young versus old	3.0 year
Otten et al., 2013	Netherlands	Single-center cohort (prospective)	3714, 708	PCI (Primary)	In young versus old and men versus women	1.0 year
Roth et al., 2016	Austria	Multicenter cohort (retrospective)	2046, 545	PCI (Elective)	In different ages	5.5 year
Xu et al. (LOTUS), 2018	China	Single-center cohort (prospective)	1792, 486	CABG versus PCI versus MT (TVD)	–	6.8 year
Li et al. 2017	China	Matched single center cohort (retrospective)	2018, 142	CABG versus PCI (DM)	–	5.0 year
Fu et al. 2018	China	Matched single-center cohort (retrospective)	1920, 227	PCI (STEMI)	In men versus women	1 and 3 year

MI: Myocardial Infraction; Revascularization: consist of coronary artery bypass grafting and percutaneous coronary intervention; CABG: Coronary Artery Bypass Grafting; PCI: Percutaneous Coronary Intervention; MT: Medical Therapy; HF-LVSD: Heart Failure and Left Ventricular Systolic Dysfunction; TVD: Three Vessel Disease; DM: Diabetes Mellitus; STEMI: ST Elevation Myocardial Infraction

**Table 5:** Other end points in CAD patients under 60 years old at different follow-ups

Study name	Follow-up (year)	MI (%)	Stroke (%)	Re-revascularization (%)
CABG	7.9	290/3523 (8.2%)	174/3523 (4.9%)	343/3523 (9.7%)
PCI	–	469/9123 (5.1%)^€^	92/5042 (1.8%)^€€^	212/2434 (8.7%)^€€^
MT	5.7	94/1093 (8.6%)	28/1093 (2.6%)	45/400 (11.3%)^€€€^

€: after 4.7 years of follow-up; €€: after 8.4 years of follow-up; €€€: after 6.8 years of follow-up; PCI: percutaneous coronary intervention; CABG: coronary artery bypass graft; MT: medical treatment

The follow-up of eligible studies was somewhat different; as one-, three-, five-, and seven-year were common in studies that reported CABG and PCI. Also, MT is reported in COURAGE and LOTUS. There were only 5- and 7-year follow-up [14, 15]. Only in one study, 10-year follow-up after revascularization was reported, therefore, this period was omitted in probabilities.

Our analysis is based on pooled data from 16,410 patients with PCAD and provides strong evidence about the death probabilities of CAD patients under 60 years old. The probabilities obtained were highly comprehensive. The age range of PCAD patients is considered slightly wider to compensate for insufficient studies. For this reason, we considered COURAGE as one of the eligible studies, since its maximum age was 65 years. In other eligible studies, the maximum age was 60 years or less.

The study population includes almost any type of baseline characteristic. Our baseline characteristics are close to other similar studies, except age [2, 6, 8, 9]. Like meta-analyses done so far, the history of stroke was less than 5%, the history of hypertension was about 50%, and the hyperlipidemia was about 40%. Males make up a significant proportion of the population and were more than 80% (13,181/15,785; 83.5%). This ratio was almost the same in the all procedures. Contrary to our expectations, no high family history was observed in eligible studies; as 32% of the population had a family history and varied from 10 to 50% in Fu et al. and COURAGE, respectively [15, 22]. This conveys the importance of environmental factors.

In most meta-analyses, mortality statistics are limited to elderly patients [2, 7–9]. Except for Hlatky et al., no meta-analyses reported mortality under 60 years of age [6]. According to Hlatky, during 5·9 years after CABG and PCI, overall mortality was 5.5% and 5.0%, respectively, in patients less than 55 years old. Given in proportion of the length of the follow-up, these are close to our probabilities. This seems logical; since all of the Hlatky population were multi-vessel and follow-up was longer [6].

Also, Hltaky’s results about CAD less than 55 years old are brief and insufficient. It is limited to revascularization, as MT is omitted. Most of the findings are related to more than 65 years old. The population did not have comprehensive clinical characteristics (because limited to multi-vessel patients), and it has not examined the mortality rates of patients under 60 years old in detail. Therefore, Hlatky probabilities will not be suitable for inserting the cost-effectiveness study related to CAD patients less than 60 years old [6]. Other meta-analyses that performed so far are almost the same. [2, 7–10, 12, 13].

In our meta-analysis, the overall mortality probabilities of CAD patients under 60 years old based on different follow-ups and each procedure were reported, which is unique. Probabilities are widely generalizable. The first reason is that the study population is sufficient (n = 16,410) which almost was twice similar studies (Hlatky: 7812; Beetle: 7798 people). Second, eligible studies covered almost any baseline characteristic.

In parts B and C which are the side parts of the research, comparisons were made based on the type of treatment or the baseline characteristics. Some results of these parts (especially Part B) do not seek to prove which treatment is appropriate for PCAD patients. For example, Fig. 5 is provided for more informational purposes and is not a criterion for clinical decision-making. Because it shows the comparisons without modifying the clinical-diagnostic characteristics of the population. In contrast, Figs. 6, 7, 8, 9, 10 and 11 can be the subject of a separate study. Because it has compared procedures based on some specific characteristics.

Figures 9 and 11 show mortality and MI after PCI in male compared with female PCAD patients, respectively. Only two studies (Fu et al. and Otten et al.) compared this comparison between men and women under 60 years old. Patients in both studies are STEMI. Therefore, the population of Figs. 9 and 11 is homogeneous, and it can be strongly acknowledged that mortality after PCI is higher in women than men with PCAD (Fig. 9). In terms of MI, no difference was observed between these groups (Fig. 11).

Figure 8 is obtained from four studies. The population of STICH and Otten were HF-LVSD^1^ and STEMI, respectively, and the populations of the two other studies (COURAGE and Roth) were stable angina. As shown in Fig. 8, the mortality after PCI was higher in younger patients than older patients in all studies. So, although the populations are heterogeneous, the results are homogeneous. This means that mortality after PCI in young CAD patients (PCAD) is much lower than that in the elderly; and it does not matter if the patients were HF-LVSD, STEMI or stable angina. Figures 7 and 10 also follow the rule of population heterogeneity and homogeneity of results, but there is no difference between old and young in terms of mortality and MI; that it does not matter if the patients were STEMI or stable angina.

In general, the results of part A showed that CABG had the lowest mortality, despite the differences in baseline characteristics. Deb et al. also found no difference between CABG and PCI in their studies. However, it should be noted that our patients are younger [8].

Our comparisons based on the type of treatment or the type of baseline characteristic may seem pointless. Because many studies have been done to compare treatments and most have preferred revascularization to medication. But it is better to note that the limitations of studies are the high average age. Parts B and C have answered the gap of these studies (i.e., comparisons at younger ages). Therefore, the results may be similar and somewhat repetitive, but the age range and consequently the average age are significantly different from the rest. For example, the median age of the Hlatky meta-analysis was 61 years (IQR 53–67), and the mean age of the Fulcher meta-analysis was 62.9 ± 6 years. While our age range is 20–60 years, the mean age is 51.2 ± 6. This mean age is 10 years less than the common CAD studies.

On the other hand, our target age range was the biggest constraint for selecting studies. There were insufficient studies in this age range. Therefore, we considered randomized and observational studies. The main limitation of this study was the lack of meta-analysis of other end points such as MI, stroke and re-revascularization. It is enough to report their raw percentages in Table 5. This table shows 8.2%, 5.1%, and 8.6% myocardial infarction in PCAD patients during 7.9, 4.7, and 5.7 years after CABG, PCI, and MT, respectively. These will not be used in cost-effectiveness study, since there are drawbacks to other end points, such as 1. Among eligible studies, the number of studies that reported other end points was very low 2. The follow-ups of the other end points were inconsistent 3. There was no uniform definition of re-revascularization in the eligible studies. Therefore, a meta-analysis of other end points was omitted.

Specific baseline characteristics of some eligible studies have enhanced the generalizability of our results. It has made it possible to compare patient mortality based on the same treatment, which is discussed in Part C. Many studies have been done on CAD outcomes so far. The baseline characteristic differences between some of our eligible studies reflect that PCAD studies are not sufficient. Therefore, it is recommended more studies be done on CAD outcomes under 60 years old in the future. In the end, the death probabilities obtained play an important role in determining the survival rate and determining the years of life obtained after three strategies. Then, these values will be used to determine the cost-effectiveness of procedures in CAD patients less than 60 years old.

## Data Availability

Not applicable.

## Abbreviations

CAD: Coronary artery disease
CABG: Coronary artery bypass graft
PCI: Percutaneous coronary intervention
MT: Medical therapy
MACCE: Major adverse cardiac and cerebrovascular events
CABG and PCI: Revascularization

## Footnotes

1 Heart Failure and Left Ventricular Systolic Dysfunction.
